# Clinical Applications of Artificial Intelligence in Teleorthodontics: A Scoping Review

**DOI:** 10.3390/medicina61071141

**Published:** 2025-06-25

**Authors:** Alessandro Polizzi, Sara Serra, Rosalia Leonardi, Gaetano Isola

**Affiliations:** Department of General Surgery and Medical-Surgical Specialities, University of Catania, 95124 Catania, Italy

**Keywords:** artificial intelligence, clear aligner therapy, deep learning, dental monitoring, remote monitoring, teleorthodontics

## Abstract

*Background and Objectives*: To systematically map and evaluate the current literature on the application of artificial intelligence (AI) in teleorthodontics, focusing on clinical use, technological approaches, outcomes, and limitations. *Materials and Methods*: A scoping review was conducted following a formal and recognized methodological framework. Three databases (PubMed, Scopus, Web of Science) were searched until 30 April 2025. Studies were included if they reported original data on AI applications in orthodontic remote monitoring or virtual care. Data extraction focused on study design, type of AI, clinical setting, reported outcomes, and main findings. *Results*: Nine studies met the inclusion criteria. Most research focused on the use of the Dental Monitoring™ (DM) system, which employs deep learning algorithms to analyze intraoral scans captured via smartphones. Reported benefits included reduced in-office visits (up to 33%), accurate 3D tracking of tooth movement, improved hygiene compliance, and high patient engagement. However, significant variability was observed in the repeatability and precision of AI decisions, especially in GO/NO-GO aligner progression instructions. One study explored an alternative system, StrojCHECK™, based on a decision tree algorithm, showing improved compliance with personalized feedback. *Conclusions*: AI-powered teleorthodontic systems show potential to enhance treatment efficiency and patient engagement, particularly in aligner therapy. However, their current clinical application remains narrowly focused on commercial monitoring platforms, with limited validation and transparency. This review highlights the early stage of real-world AI integration in orthodontics, underlining the need for independent validation, broader applications beyond monitoring, and robust ethical frameworks. In this context, AI should be used as a complementary tool, never a substitute, for clinical judgment.

## 1. Introduction

Artificial Intelligence (AI) is playing an increasingly pivotal role in the digital transformation of healthcare [1]. In orthodontics, AI-driven systems are rapidly emerging as tools that augment traditional workflows and open the door to novel modes of treatment delivery. Machine learning and deep learning algorithms, subfields of AI, enable systems to perform complex tasks such as image recognition, predictive analytics, and pattern detection, which are now being applied to various aspects of orthodontic care, including diagnosis, treatment planning, and progress assessment [2,3,4].

In parallel, the field of teleorthodontics has gained substantial momentum, particularly in response to growing demands for efficiency, accessibility, and convenience in patient care. Teleorthodontics refers to the remote delivery or monitoring of orthodontic services through digital means [5]. While early applications were limited to virtual consultations or review of static images, recent advances now enable dynamic, asynchronous interactions where clinical assessments are performed remotely through AI-analyzed images or video scans captured by the patient [6]. Moreover, among the various dental disciplines, orthodontics presents a unique context for the application of AI in remote care. Unlike other fields where treatment is often episodic or short-term, orthodontic therapy is typically long in duration, requires continuous adjustments, and depends heavily on patient compliance. These characteristics could have the potential to make teleorthodontics particularly suited for AI integration, especially for remote monitoring and behavioral support.

The COVID-19 pandemic served as a powerful catalyst for the adoption of remote technologies in dentistry. With widespread clinic closures and restrictions on in-person visits, orthodontists were compelled to maintain patient continuity using digital platforms [7]. In this context, AI-enabled teleorthodontic tools provided an effective alternative for clinical oversight and patient engagement, bridging the gap between lockdown restrictions and ongoing orthodontic care [8].

One of the most prominent commercial systems in this domain is Dental Monitoring™ (DM), which utilizes convolutional neural networks (CNNs) to analyze patient-submitted intraoral images or videos. Through this process, DM provides clinicians with automated updates on treatment progression, hygiene status, and appliance integrity. The platform also offers GO/NO-GO functionality to evaluate aligner fit and determine readiness for the next treatment step without the need for in-person appointments [9]. These AI-driven platforms do not merely enhance clinical convenience—they also transform the patient experience. Patients are empowered to participate actively in their treatment journey, submitting regular scans from home using smartphone applications and receiving timely feedback on their progress. This interaction contributes to higher compliance, improved motivation, and potentially more favorable outcomes [10]. Moreover, AI-based systems in teleorthodontics have been applied to track tooth movement and to detect loss of tracking, unwanted movement patterns, or hygiene-related issues such as plaque or gingivitis [11].

Despite the enthusiasm surrounding these tools, important concerns should be addressed. One of the key challenges relates to the reliability and clinical agreement of AI assessments with standard diagnostic methods [12]. Moreover, ethical considerations arise in the context of AI delegation, data privacy, and the potential loss of the human touch in patient care [13]. In addition, many of the systems currently in use are proprietary and operate as “black boxes,” where the internal mechanisms of decision-making are not fully transparent to clinicians. This opaqueness can hinder informed clinical judgment and limit the ability to contest or validate AI-generated recommendations [14].

In light of the recent emerging developments and challenges, this scoping review aimed to map and describe the current clinical applications of AI in teleorthodontics, with particular attention to the types of systems used, their technological features, reported outcomes, and the limitations or gaps highlighted in the available literature.

## 2. Materials and Methods

### 2.1. Research Strategy and Study Design

This scoping review followed the methodological framework proposed by Arksey and O’Malley [15], and further refined by Levac et al. [16], which guided each phase of the review process, including study identification, selection, data charting, and synthesis. The research question was as follows: What are the current clinical applications and reported outcomes of artificial intelligence in teleorthodontics? A comprehensive literature search was performed on 6 May 2025, across three major electronic databases: PubMed, Scopus, and Web of Science, covering all records up to 30 April 2025. The search strategy was designed to retrieve studies combining concepts related to artificial intelligence (AI) and remote orthodontic practice. The following Boolean search string was used (with syntax adapted per database): (“teleorthodontics” OR “remote monitoring” OR “dental monitoring” OR “teledentistry cephalometrics” OR “smart application”) AND (“artificial intelligence” OR “AI”). The search strategy was developed iteratively and adapted to each database to ensure comprehensive coverage.

### 2.2. Inclusion and Exclusion Criteria

To ensure relevance and focus, the eligibility criteria were defined based on the PICOS framework:

- Population (P): Orthodontic patients of any age undergoing monitoring or treatment in a remote or virtual context.
- Intervention (I): Applications clearly focused on AI, including machine learning, deep learning, and neural networks.
- Comparison (C): Not applicable.
- Outcomes (O): Any reported clinical, technical, or usability-related outcomes related to AI in a teleorthodontic context.
- Study design (S): Original research articles, case series, or pilot studies.

Studies were included if (1) they focused on the use of artificial intelligence in orthodontics within a remote, virtual, or telemonitoring setting, (2) if published in peer-reviewed journals, and (3) if available in English full-text. Review articles, editorials, letters to the editor, theses, and commentaries were excluded. Moreover, original studies not specifically involving orthodontics (e.g., general dentistry without orthodontic context), not explicitly involving any form of AI, or not available in full-text were not considered in this scoping review.

### 2.3. Selection of Studies and Data Extraction

All records were first analyzed for duplicate removal and initial organization. The selection process followed a two-step screening approach: (1) title and abstract screening of the records, and (2) full-text assessment to evaluate if the articles fully met the inclusion criteria. Both steps were independently conducted by two reviewers. Discrepancies were resolved through discussion or by consulting a third reviewer. The following data were extracted and synthesized from the included studies: author(s), year, study design and population, type of AI used, orthodontic application and setting, reported outcomes, and key findings.

In line with established scoping review methodology, no formal risk of bias assessment was performed, as the objective was to map and characterize the existing evidence rather than evaluate the methodological quality of the included studies.

## 3. Results

**Figure 1:**
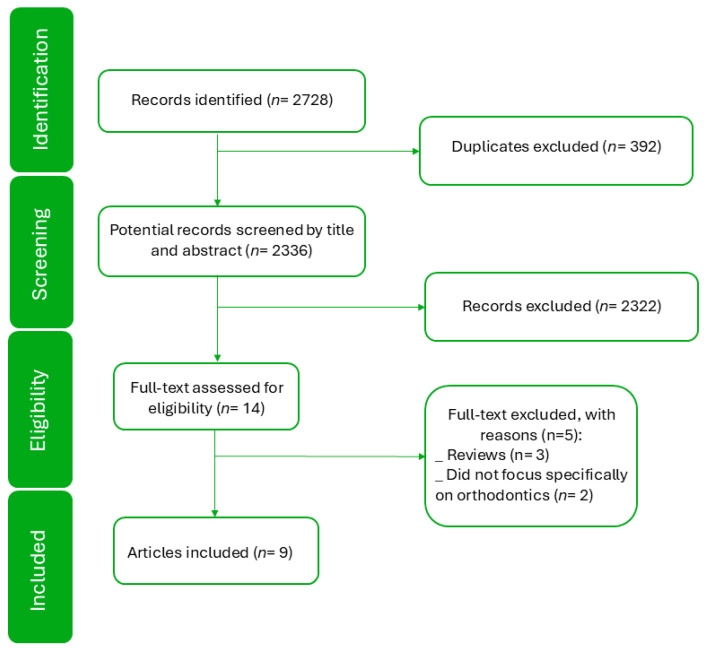
Study selection flow diagram.

The initial search yielded a total of 2728 records across the three databases: 436 from PubMed, 1680 from Scopus, and 612 from Web of Science. Following the removal of duplicates and the two-step screening process, nine articles met the inclusion criteria and were included in the final analysis (Figure 1).

Table 1 summarizes the key characteristics of the included studies, providing an overview of the articles, their aims, study designs, the type of AI utilized, the orthodontic application and setting, reported outcomes, and main findings.

The included studies demonstrate a range of applications of artificial intelligence in teleorthodontics. A significant portion of the research focuses on remote monitoring using the Dental Monitoring™ system. Arqub et al. (2023) [17] quantified real-time tooth movement during space closure, demonstrating the accuracy of Dental Monitoring™ scans and observing that tooth movement is most pronounced in the initial phase after activation. Caruso et al. (2021) [18] highlighted the system’s utility for monitoring complex orthodontic movements and aligner fit, especially during the COVID-19 lockdown. Other studies, such as Homsi et al. (2023) [21], validated the accuracy of 3D digital models generated by the Dental Monitoring™ system. Snider et al. (2023, 2024) [22,23] evaluated the effectiveness of AI-based active reminders in improving oral hygiene during fixed orthodontic treatment, showing a statistically significant reduction in oral hygiene indices in the AI-monitored group.

Beyond Dental Monitoring™, Thurzo et al. (2021) [25] investigated the impact of AI within the StrojCHECK™ app, focusing on treatment coaching and its influence on patient compliance.

Table 2 provides a focused overview of the AI systems identified in the included studies. Dental Monitoring™ is characterized by its use of deep learning and knowledge-based algorithms to facilitate remote monitoring through a smartphone app and ScanBox, providing features like automated communication and “GO/NO-GO” notifications for aligner progression in Clear Aligner Therapy (CAT). StrojCHECK™ is presented as a free app that utilizes decision tree algorithms for treatment coaching and monitoring patient compliance. While StrojCHECK™ employs sophisticated algorithms, the core app does not use AI in the same way as Dental Monitoring™. In particular, it does not utilize AI for direct analysis of patient-submitted data but instead uses decision trees to guide patient behavior and treatment adherence.

## 4. Discussion

Multiple studies have supported the clinical utility of AI-driven remote monitoring in improving patient engagement and treatment efficiency. For instance, Hansa et al. [20] reported that DM allowed earlier intervention, reduced the number of face-to-face visits by up to 33%, and improved hygiene monitoring and compliance across a range of orthodontic indications, including aligners and fixed appliances. Similarly, Arqub et al. [17] demonstrated that tooth movement is most pronounced during the initial phase after activation and that DM can accurately capture small changes in displacement, with a maximum deviation of 0.05 mm compared to digital reference models.

However, despite the promising results, some limitations and challenges remain. Ferlito et al. [19] questioned the reliability of DM’s GO/NO-GO feature, which is used to determine readiness for aligner progression. Their study showed that, although scan repeatability was 83.3%, there was a 0% agreement on the specific teeth identified as problematic between consecutive scans, raising concerns about the internal consistency and reproducibility of the algorithm’s outputs. The authors also pointed out that some AI decisions lacked clinical correlation, with up to 2 mm and 8° of discrepancy in patients receiving the same GO or NO-GO instruction, which is clinically significant. Moreover, as noted by Snider et al., even though DM’s hygiene alerts showed high specificity, their sensitivity for detecting gingivitis or recession was low, implying that AI is more effective at ruling out rather than detecting conditions [23].

In this context, as previously mentioned, the interpretability of these AI outputs remains a debated issue. Unlike conventional diagnostic tools, deep learning models function as “black boxes,” meaning clinicians often cannot discern the reasoning behind a specific output [26]. This lack of transparency can impair trust and make it difficult to integrate AI suggestions into complex clinical decisions, especially when results contradict clinical judgment.

In addition, the evolution of AI-assisted care could also imply a substantial future change in team dynamics. The role of dental assistants and nurses could shift toward remote patient engagement, data triage—such as scan alerts, compliance warnings, prioritize cases requiring clinical attention, communicating relevant findings to the orthodontist—and communication. In this regard, Surovková et al. [24] documented how 98% of Invisalign patients in their practice used DM, resulting in a substantial redistribution of workload among staff. Assistants were more involved in managing digital communication, flagging issues from AI reports, and instructing patients based on remote findings. This transformation of traditional roles may bring both opportunities and challenges: while it enhances efficiency and personalization, it also raises questions about training, delegation, and liability in case of AI errors [27].

Interestingly, not all systems relied on deep learning. The StrojCHECK™ platform, explored by Thurzo et al. [25], uses a decision tree-based engine integrated within a mobile coaching app. Unlike systems such as Dental Monitoring™, which process intraoral images through neural networks, StrojCHECK™ focuses on behavioral support. It does not analyze patient-submitted scans or assess tooth movement; rather, it provides automated motivational feedback based on patient interaction patterns. In their study, the authors observed improved patient compliance following the AI-based update, particularly among female patients, as reflected by a reduction in non-tracking (NO-GO) scan reports.

Overall, from a clinical perspective, AI-based remote monitoring appeared to be a valuable adjunct to traditional orthodontic care, particularly in the context of clear aligner therapy. According to several of the included studies, it increased the frequency of patient monitoring and enabled more detailed tracking of treatment progress. These systems empower patients to engage more actively in their own care and may allow orthodontists to dedicate more time to complex decision-making.

However, their limitations in diagnostic sensitivity, consistency, and explainability must be acknowledged. Therefore, future research should prioritize external and internal validation of AI systems and head-to-head comparisons with clinician assessments. In addition, ethical concerns should also be addressed, especially around patient consent, data protection, and the potential for overreliance on automation. The implementation of AI-based systems in teleorthodontics is not free from important ethical and regulatory issues [13]. Data privacy and patient consent are central, particularly when sensitive health data are transmitted through third-party platforms or stored on cloud-based infrastructures [28,29]. The use of proprietary algorithms, often functioning as “black boxes”, limits clinicians’ ability to understand or challenge AI-generated recommendations, raising questions about professional accountability and medico-legal liability [30]. Furthermore, current regulatory frameworks for medical devices and digital health tools do not yet fully address the specific challenges posed by autonomous or semi-autonomous AI systems [31]. Establishing clear standards for validation, transparency, data governance, and clinician oversight is essential to ensure ethical and safe adoption of AI technologies in orthodontics.

Another limitation of the current body of evidence is the exclusive reliance on commercial platforms, particularly Dental Monitoring™, for AI-based remote monitoring. No clinical studies using open-source or non-commercial AI systems were identified. This highlights a gap in the literature and emphasizes the need for future research to explore independent or open-source AI models that could improve transparency, reduce costs, and enhance accessibility. Nevertheless, this review has notable strengths: It is, to our knowledge, the first to systematically map the current landscape of clinically implemented AI systems for teleorthodontic monitoring, using a structured and replicable framework. Furthermore, by focusing exclusively on real-world patient applications, it provides a clinically relevant synthesis of what is currently achievable in practice. However, due to the dominance of a single commercial platform (Dental Monitoring™), and the narrow scope of AI functionalities evaluated, generalization to other technologies or broader clinical settings remains limited. Further studies using diverse systems, standardized methodologies, and long-term patient outcomes are needed to strengthen the evidence base and inform clinical adoption.

Moreover, one peculiar aspect evidenced by the present scoping review is the heterogeneity of study designs among the included articles. The literature spans from single-case reports and descriptive series to prospective clinical trials, often with small and variable sample sizes. As such, the risk of bias in design and interpretation cannot be excluded. While this diversity reflects the emerging and exploratory nature of AI applications in teleorthodontics, it limits the possibility of drawing direct comparisons or generalizable conclusions. The outcomes reported were often limited to descriptive or process-based indicators, such as scan frequency, GO/NO-GO status, or patient engagement, rather than objective clinical endpoints. This limits the strength of the conclusions and the ability to assess clinical effectiveness. For this reason, this review did not attempt to assess relative effectiveness but rather to map the range of clinical scenarios and AI functions being studied. Future research should aim to standardize study designs, use larger and more representative samples, and prioritize comparative outcomes where possible.

In addition, although AI has become a rapidly expanding area in dentistry, the number of studies meeting the inclusion criteria was notably limited, reflecting the current maturity of the field. The present review focused specifically on clinical studies that implemented AI-driven systems for remote orthodontic monitoring in real patients. As such, experimental designs without patient interaction, and papers focused on other dental disciplines or non-AI-based telemonitoring systems have been excluded. The resulting number of included studies, while seemingly modest, highlights a key issue: despite the technological hype surrounding AI, its real-world clinical adoption in teleorthodontics remains at an early stage. This scarcity of robust evidence emphasizes the need for independent, well-designed, and clinically integrated studies to assess the long-term value, safety, and cost-effectiveness of AI in routine orthodontic care.

In this regard, while current AI applications in teleorthodontics focus primarily on monitoring treatment progress and patient compliance, broader telemedicine frameworks may suggest promising yet underexplored directions. One particularly relevant avenue is the development of AI-assisted triage systems, which have been successfully integrated in various areas of medical teleconsultation to support clinical prioritization, risk stratification, and resource optimization [32]. Orthodontics is inherently well-suited for such systems, as it begins with a highly structured collection of diagnostic data, including extraoral and intraoral photographs, radiographs, cephalometric analyses, and 3D digital models, many of which are already acquired in digital format. These standardized datasets create ideal conditions for machine learning models to perform preliminary assessments. In this context, an AI-based triage system could help clinicians by automatically classifying malocclusions by severity, detecting skeletal discrepancies or airway concerns, identifying potential contraindications, or even suggesting possible treatment pathways (e.g., interceptive vs. comprehensive therapy, aligners vs. fixed appliances). Such tools could not only increase diagnostic efficiency but also improve access to care by enabling asynchronous consultation, early case sorting, and more appropriate allocation of specialist time. Importantly, these systems would not aim to replace clinical expertise but to act as intelligent assistants, enhancing workflow, informing early decisions, and reducing bottlenecks in patient intake. Future research should explore the feasibility, validation, and clinical integration of AI triage tools specifically adapted for orthodontics, without neglecting potential ethical concerns and drawing inspiration from proven models in broader telemedicine settings.

## 5. Conclusions

This scoping review identified a limited but growing body of evidence supporting the use of AI-powered systems for teleorthodontic monitoring. Reported benefits include improved efficiency, patient compliance, and workflow redistribution, especially in the context of aligner therapy. Nonetheless, current applications are largely confined to a single commercial platform and focused on remote monitoring, with significant gaps in diagnostic reliability, generalizability, and clinical transparency. AI systems should be viewed as supportive tools, not replacements for orthodontic expertise. Future research should prioritize independent validation, real-world outcome studies, and the development of AI applications capable of supporting earlier phases of care, including diagnosis, triage, and treatment planning. This will be essential to move from technological potential to clinically meaningful integration.

## Figures and Tables

**Table 1:** Characteristics of the included studies.

Article	Aim	Study Design and Population	Type of AI	Orthodontic Application and Setting	Reported Outcomes	Main Findings
Arqub SA et al. 2023 [17]	To quantify real-time orthodontic tooth movement and evaluate the accuracy of DM scans compared to iTero models during space closure	Prospective pilot study; 12 fixed appliance patients (mean age 20.1) undergoing canine retraction	Deep learning via convolutional neural network; Dental Monitoring™ system	Remote monitoring of tooth movement using patient-acquired video/photo scans every 4–5 days	Tooth movement peaked in first 4–5 days; rate declined thereafter; DM scans had max deviation of 0.05 mm from iTero; molars showed anchorage loss	Tooth movement occurs mostly early after activation; DM is accurate for 3D monitoring; first detailed study on short-interval displacement tracking using AI
Caruso S et al., 2021 [18]	To present two cases using the Dental Monitoring (DM) system to monitor orthodontic treatment.	Case report of two patients: a growing patient with a retained upper canine and an adult patient with an upper lateral incisor crossbite. Both treated with aligners.	Deep learning via convolutional neural network; Dental Monitoring™ system	Remote monitoring of orthodontic treatment through patient-taken intra-oral pictures using a smartphone and ScanBox. Monitoring aligner fit and retention.	Successful monitoring of complex movements and aligner fit. Effective during COVID-19 lockdown.	DM system is a promising method for remote monitoring, improving doctor-patient interaction. Useful in complex cases and during disruptions like the COVID-19 pandemic.
Ferlito T et al., 2023 [19]	To assess the capacity of AI-based remote monitoring to determine readiness to progress to the next aligner (GO/NO-GO) and measure tooth position discrepancies in clear aligner therapy	Prospective study; 30 aligner patients scanned twice for repeatability; 24 additional aligner patients at final stage used for 3D comparison	Deep learning via convolutional neural network; Dental Monitoring™ system	Remote monitoring in clear aligner therapy (AI-based assessment of tracking and progression readiness)	83.3% repeatability in GO/NO-GO, but 0% agreement on which/how many teeth had tracking issues; max discrepancies up to ~2 mm and ~8° in multiple axes	Significant variability in AI decisions; lack of consistency in instructions and discrepancy with actual 3D tooth positions raise concerns about reliability.
Hansa I et al., 2021 [20]	To discuss the clinical applicability and rationale of AI-driven remote monitoring (AIRM) in orthodontics through a case series	Descriptive case series; clinical examples from aligner and fixed appliance patients	Deep learning via convolutional neural network; Dental Monitoring™ system	Monitoring of aligner fit, hygiene, attachments, bracket failures, RME, eruption, retention; remote setting with smartphone app and Doctor Dashboard	Reported reduction in visits (up to 33%), early detection of issues (e.g., aligner tracking loss, gingival problems), improved compliance	AIRM allows earlier intervention, fewer in-office visits, improved doctor–patient communication; promising for aligners and fixed appliances; some limitations include cost and patient preference for in-office care.
Homsi K et al., 2023 [21]	To compare the accuracy and validity of 3D digital models generated by AI-based Dental Monitoring™ vs. iTero scans during fixed orthodontic treatment	In vivo longitudinal study; 24 patients (14–55 years) tracked for 13.4 months using DM and iTero scans	Deep learning via convolutional neural network; Dental Monitoring™ system	Remote monitoring during fixed orthodontic treatment; reconstruction of 3D dental models	No clinically significant difference in 3D model accuracy between DM and iTero at any time point; deviations remained within clinically acceptable limits	AI-based DM technology can reconstruct 3D models and track tooth movement accurately enough for clinical orthodontic use.
Snider V et al., 2023 [22]	To evaluate if AI-based active reminders from Dental Monitoring™ improve oral hygiene during fixed orthodontic treatment	Prospective clinical study; 24 patients monitored with DM for 10–13 months vs. 25 control patients for 3–5 months	Deep learning via convolutional neural network; Dental Monitoring™ system	Remote monitoring of oral hygiene; smartphone app notifications and periodic assessments	At T5: DM group had lower OPI (2.00) and MGI (1.60) vs. control (OPI: 2.75, MGI: 2.63); statistically significant differences (*p* < 0.05)	AI reminders helped maintain better hygiene vs. controls; hygiene worsened over time despite reminders; DM group showed plateau in plaque scores but not gingival index; scan adherence declined over time
Snider V et al., 2024 [23]	To clinically evaluate the accuracy of Dental Monitoring™’s (DM’s) AI image analysis and notification algorithm for detecting plaque, gingivitis, and recession during orthodontic treatment	Prospective clinical study; 24 orthodontic patients, 232 clinical timepoints	Deep learning via convolutional neural network; Dental Monitoring™ system	Remote monitoring of oral hygiene during treatment with fixed appliances	Sensitivity: 0.53 (plaque), 0.35 (gingivitis), 0.22 (recession); Specificity: 0.94–0.99; Accuracy: 0.49–0.72	High specificity but low sensitivity; DM tends to underreport oral hygiene issues; useful to confirm absence, but not presence, of problems.
Surovková J et al., 2023 [24]	To present a new AI-powered orthodontic workflow and evaluate its impact on dental assistants/nurses’ roles, patient monitoring, and treatment efficiency	Observational, practice-based preliminary report; 372 DM users and 192 non-DM controls in Invisalign treatment over 3 years	Deep learning via convolutional neural network; Dental Monitoring™ system	AI-assisted remote monitoring of clear aligner therapy; emphasis on staff workflow, aligner tracking, hygiene, and patient communication	98% of Invisalign patients in the practice used DM; staff workload shifted to remote communication and data triage; improved perception of early error detection and task delegation)	AI transformed roles of nurses/assistants—less chairside work, more communication and data triage; DM enhances monitoring but needs human validation; AI supports personalization, but raises ethical/legal issues.
Thurzo A et al., 2021 [25]	To evaluate the impact of an AI-driven update to the StrojCHECK™ orthodontic coaching app on patient compliance and clinical outcomes via remote monitoring	Pre-post observational study; 86 aligner patients (12–68 years) using the StrojCHECK™ smart coaching app before and after its AI-based update	Decision tree AI algorithm implemented in StrojCHECK™ coaching app (internal engine)	Smart coaching via app + AI remote monitoring of aligner tracking via mobile app	Significant improvement in user interaction and discipline; decrease in NO-GO scans (non-tracking) in females; no age effect; GO scans unchanged	AI update led to improved compliance and clinical behavior; patient-specific AI coaching enhanced performance; clinical improvements were gender-dependent and suggest the need for personalized motivational strategies.

**Table 2:** AI teleorthodontic systems.

AI System	Algorithm Type	Characteristics	Clinical Benefit	References
Dental Monitoring™	Deep learning via convolutional neural network	Smartphone app with ScanBox for patient-acquired intraoral images, enabling detailed remote monitoring of aligner fit and tooth movement; Doctor Dashboard for remote monitoring; automates communication and provides “GO/NO-GO” notification based on AI analysis of aligner fit and tracking.	Improved treatment efficiency, reduced need for some in-office visits, enhanced aligner tracking and treatment control.	[17,18,19,20,21,22,23,24]
Decision tree AI algorithm implemented in StrojCHECK™	Decision tree algorithm	Free smartphone app for orthodontic patients and doctors, designed to enhance patient compliance and adherence to treatment protocols; Monitors patient activities and compliance; Server back-end for doctors for statistical data processing, providing clinicians with valuable insights into patient behavior and progress. Does not utilize AI in the core app functionality but decision tree algorithms were integrated to guide patient behavior.	Increased patient compliance, better communication, objective monitoring of patient behavior.	[25]

## Data Availability

The data underlying this article are available in the article.
